# Reassessing the interpretation of oxidation–reduction potential in male infertility

**DOI:** 10.1530/RAF-21-0005

**Published:** 2022-03-18

**Authors:** Fabien Joao, Cyntia Duval, Marie-Claire Bélanger, Julie Lamoureux, Cheng Wei Xiao, Senem Ates, Moncef Benkhalifa, Pierre Miron

**Affiliations:** 1 Fertilys Fertility Center, Laval, Quebec, Canada; 2 Centre de Recherche du Centre Hospitalier de l’Université de Montréal (CRCHUM), CHUM Research Center, Montreal, Quebec, Canada; 3 Université Picardie Jules Verne, Médecine et Biologie de la Reproduction et Laboratoire PERITOX, CBH-CHU Amiens Picardie, Amiens, France

**Keywords:** sperm, oxidoreduction potential, DNA integrity, male infertility

## Abstract

Male Infertility Oxidative System (MiOXSYS) has been proposed as a rapid and promising technology for the evaluation of sperm oxidative stress. In this case–control study, 134 men with normal sperm parameters (NSP) and 574 men with abnormal sperm parameters (ASP), according to the World Health Organization sperm assessment references values established in 2010, were enrolled. Conventional sperm parameters were evaluated in all patients. Sperm static oxido-reduction potential (sORP) was assessed using the MiOXSYS. Sperm DNA integrity was measured in 604 patients. To ensure that sperm concentration was not a confounding factor in the sORP index ratio, sperm and seminal fluid sORP from 57 randomly selected additional patients were also measured using the MiOXSYS. sORP index (mV/10^6^ sperm/mL) was higher in patients with ASP and seemed to correlate with conventional sperm parameters. Although receiver-operating characteristic analysis revealed that a sORP index cut-off of 0.79 could differentiate normal from ASP with 57.7% sensitivity and 73.1% specificity, these values are much lower than those found in the literature. These values also need to be higher to be applicable in a clinical setting. Furthermore, absolute sORP (mV) was not different in the presence or absence of spermatozoa. sORP index relationships with sperm parameters seem rather be due to sperm concentration, denominator of the sORP index ratio. The establishment of a reliable method using the absolute sORP value, independent of sperm concentration, needs to be addressed. Other oxidative stress biomarkers could be used to validate this method.

**Lay summary:**

The World Health Organization (WHO) has recognized that oxidative stress may have a role in male infertility. Oxidative stress happens when there is an imbalance between the production of molecules containing oxygen and the antioxidants, molecules that neutralize the molecules containing oxygen. The molecules containing oxygen can cause damage to sperm DNA. This damage can be measured using a particular index and this study looked at whether the concentration of the sperm sample might have an impact on results and suggests this should be taken into consideration by clinicians and researchers.

## Introduction

Classical semen assessment consists of the analysis of standard descriptive sperm parameters such as concentration, motility and sperm morphology (World Health Organization 2010). However, this analysis is limited and does not allow the detection of abnormalities at the molecular level, such as DNA damage, protamines/histones ratio and DNA methylation dysregulation. Yet, it has been shown that sperm DNA damage is associated with reduced embryo quality (Tomsu *et al.* 2002) and reduced pregnancy rates during assisted reproduction treatments (Benchaib *et al*. 2003, Henkel *et al.* 2004, Bungum *et al*. 2007, Chen *et al.* 2019, Deng *et al.* 2019).

Recent studies have shown that oxidative stress could be involved in male infertility. Results showed that the seminal plasma of infertile men is more likely to contain high levels of reactive oxygen species (ROS) and lower concentrations of antioxidants (Pasqualotto *et al.* 2000, Chao *et al.* 2018). ROS are produced during the metabolism of oxygen and can be derived from certain chemical reactions (Ullah Khan & Wilson 1995). At low concentrations, ROS are involved in cellular processes, such as sperm capacitation (Jin & Yang 2017), but an increase in their production or a defect in their elimination may have a detrimental effect on cell metabolism (Aitken 2017). Oxidative stress is then characterized by an imbalance between the production of ROS and the ability of the cell to eliminate them. Such imbalance can lead to lipid peroxidation, DNA damage and apoptosis (Griveau & Le Lannou 1997, Aitken *et al.* 2003, Aitken 2017).

ROS are usually measured directly or indirectly through fluorescence or chemiluminescent assays (Dutta *et al.* 2019) but these tests generally measure only one aspect of oxidative stress. However, the measurement of the sORP of a sperm sample by the MiOXSYS provides an overview of the levels of oxidants and reducing agents in sperm (Agarwal *et al.* 2016). The MiOXSYS is based on the RedoxSYS© Testing System, a device used to measure sORP of blood samples from patients with brain damage. This device consists of an ultra-high impedance electrometer used with a sensor composed of two platinum electrodes and a reference Ag/AgCl electrode (Rael *et al.* 2015). A high sORP indicates an increase in oxidants and therefore oxidative stress (Rael *et al.* 2015). The absolute sORP value in mV is normalized according to sperm concentration to control for differences in sperm count and semen volume between patients (Agarwal *et al.* 2016).

In recent years, many studies have evaluated the utility of sORP in the evaluation of oxidative stress and its impact on sperm criteria during male infertility investigation (Agarwal *et al.* 2016, 2017, 2018*a*, *b*, 2019, Agarwal & Wang 2017, Ahmad *et al.* 2017, Arafa *et al.* 2018, Majzoub *et al.* 2018, 2020). These studies generally compare sORP index between patients with normal and abnormal sperm parameters (ASP) and its relationship to these parameters. Some of these studies also try to establish a sORP index cut-off to differentiate patients with normal sperm parameters (NSP) and patients withASP using receiver-operating characteristic (ROC) tests. However, none of these studies examined the possible presence of one or more confounding factors that could interfere with the interpretation of the results. Using the ratio correction method can reduce the effect of sperm concentration by normalizing all samples (Agarwal *et al.* 2016), but this method is not necessarily the most suitable in some cases (Karp *et al.* 2012).

The aims of this study were (1) to corroborate the results of the MiOXSYS by reproducing what has already been described in the literature and (2) to determine the impact of sperm concentration on the sORP index ratio by measuring the contribution of spermatozoa in the sORP value (mV).

## Material and methods

### Ethics

This study has been performed in accordance with the ethical standards as laid down in the 1964 Declaration of Helsinki as well as the ethical guidelines governing studies in which human gametes or embryos are used as materials. These ethical guidelines are issued by the Canadian Institutes of Health Research, the Natural Sciences and Engineering Research Council of Canada, and the Social Sciences and Humanities Research Council of Canada under the governance of the Assisted Human Reproduction Act, S.C. 2004. For this study, an informed and signed consent was obtained from each patient. Since this study is based on the improvement, verification or validation of a method, the University of Montreal Health Centre’s research ethics committee confirmed that it did not require an evaluation by an ethics committee.

### Patients

Seven hundred and sixty-five men going to the Fertilys reproductive health center (Laval, Québec, Canada) for a sperm analysis between October 2017 and October 2020 were enrolled in this study. Written consent was obtained for each patient. According to the WHO laboratory manual examination and processing of human semen 5th edition (Cooper *et al.*2010, WHO 2010), 608 patients were divided into two groups, depending on the normality of their sperm parameters: NSP (*n* = 134) and ASP (*n* = 574). A patient was considered ASP if at least one sperm parameter did not meet the WHO 2010 standard reference value. Patients with sperm concentration <1 million/mL were not included in this study. Fifty-seven patients were later included to investigate the contribution of spermatozoa in the establishment of the sORP value (mV). These patients were randomly chosen before their sperm analysis regardless of their age or the normality of their sperm parameters and were not included in the ASP and NSP groups.

### Semen analysis

Sperm samples were collected by masturbation following 2–7 days of abstinence. Samples were allowed to liquefy for at least 30 min at 37°C and semen analysis was performed during the next hour. A macroscopic evaluation was first performed: The Sperm Class Analyzer CASA System (Microptic S.L., Barcelona, Spain) was used to determine sperm concentration, total sperm count and motility. Sperm vitality was established using an eosin/nigrosine coloration (FertiPro, Beernem, Belgium). Air-dried smears followed by a Romanowsky coloration were prepared according to the manufacturer’s recommendations to evaluate sperm morphology using Kruger’s strict criteria (Sperm stain, Microptic S.L., Spain). A minimum of 200 sperm were examined for each test.

### Sperm oxidative stress

Sperm oxidative stress levels were evaluated using the MiOXSYS (Aytu bioscience) according to the manufacturer’s recommendations. Briefly, 30 µL of the sperm sample was loaded on the MiOXSYS sensor and inserted into the MiOXSYS analyzer. The absolute sORP values (mV) displayed on the screen were then noted and normalized with sperm concentration (10^6^ sperm/mL) to obtain the sORP index (mV/10^6^ sperm/mL).

### Seminal fluid oxidative stress

First, sperm oxidative stress was evaluated using the MiOXSYS as described in the previous paragraph. One milliliter of the sperm sample was then centrifuged at 4500 ***g*** for 10 min to obtain a sperm-free seminal fluid (Jecht & Poon 1975). A drop of seminal fluid was placed on a Makler counting chamber (Irvine Scientific, CA, USA) and analyzed on a microscope at 1000× to ensure the absence of spermatozoa. If any sperm was observed, no repeat or re-analysis was done; the sample was simply discarded. Seminal fluid oxidative stress was finally evaluated using the MiOXSYS.

### Sperm DNA fragmentation

Sperm DNA fragmentation was measured using the In-Situ Cell Death Detection Kit (Roche) in 604 patients according to the manufacturer’s recommendations. Briefly, sperm were fixed in 3.7% formaldehyde in PBS for 30 min at room temperature (RT) and washed in PBS 1×. Sperm were then permeabilized for 4 min at 4°C in a solution of pure H_2_O, sodium citrate 1% and Triton X-100. After a wash in PBS 1×, sperm were mixed with the marking solution containing the terminal deoxynucleotidyl transferase and the labeling reagent and incubated at 37°C for 45 min. DNA fragmentation was finally evaluated on 25,000 events (gated on sperm) by flow cytometry using a BD Accuri C6 (BD Biosciences, San Jose, CA, USA). Sperm were gated to eliminate debris and duplicates.

### Sperm chromatin decondensation

Sperm chromatin decondensation index (SDI) was investigated in 604 patients using aniline blue staining. During the sperm DNA fragmentation analysis, a small portion of the samples was air-dried on a slide after the permeabilization step. Smears were then immersed in a solution of aniline blue (Fisher Chemicals, Hampton, NH, USA), glacial acetic acid and pure H_2_O for 10 min at RT. Slides were finally washed 3 times in a clean water bath and air-dried. Sperm chromatin decondensation was evaluated on at least 200 sperm using a microscope at 1000×.

### Statistical analysis

Normal distribution was evaluated using the Shapiro–Wilk test. Comparisons between groups were analyzed using Student’s *t-*test when data distribution was normal and Mann–Whitney *U* test when data followed non-normal distribution. Relation between sORP index and semen parameters was established using Pearson’s correlation when data distribution was normal and Spearman’s correlation when data followed non-normal distribution. ROC curves were used to establish the best sORP index cut-off that would differentiate normal and abnormal semen parameters. Youden’s index, as defined by the higher ((sensitivity + specificity) − 1) value, was used to identify the best sensitivity, specificity (Lalkhen & McCluskey 2008, Li*et al.* 2007), positive and negative predictive values (PPV and NPV) for each sperm parameter tested. Note that prevalence was considered in the establishment of PPV and NPV since this study is of the case–control type (Steinberg*et al.* 2009). Data are presented as mean ± s.d. All data were analyzed by the scientific statistical software GraphPad Prism 8 (GraphPad Software). *P*  < 0.05 was considered significant.

## Results

Age, sperm parameters, absolute sORP (mV) and sORP index were compared between the NSP (*n* = 134) and ASP groups (*n* = 574), as shown in Table 1. Sperm concentration (*U*= 22,484; *P*  < 0.0001), total sperm count (*U*= 21,142; *P*  < 0.0001), total motility (*U*= 11,861; *P*  < 0.0001), vitality (*U*= 17,857; *P*  < 0.0001) and normal morphology (*U*= 4216; *P*  < 0.0001) were significantly reduced in the ASP group. On the other hand, age (*U*= 33,628; *P*  < 0.05), sperm chromatin decondensation (*U*= 18,605; *P*  < 0.0001) and sORP index (*U*= 23,352; *P*  < 0.0001) were significantly higher in this group. No significant difference was observed in semen volume, pH, number of immature germ cells, polymorphonuclear cells and absolute sORP (mV). Although there was a slight increase in the ASP group, sperm DNA fragmentation was not significantly different from the NSP group. It is however interesting to note that sperm DNA fragmentation index was less than 20% in more than 80% of all patients (Supplementary Fig. 1, see section on supplementary materials given at the end of this article). In addition, the sORP index was the highest when the sperm DNA fragmentation index was <5% and SDI was >20% (Supplementary Fig. 2). Sperm DNA fragmentation indexes were also distributed in a similar fashion regardless of sperm concentration but note that the higher the SDI, the lower the sperm concentration (Supplementary Fig. 3).

**Table 1:** Comparisons of age, sperm parameters and sORP in NSP and ASP patients. Comparisons between NSP and ASP patients were performed using Mann–Whitney *U* test. Note that sperm DNA fragmentation and sperm chromatin decondensation comparisons were evaluated on 604 patients when comparing the NSP (*n*= 108) and ASP groups (*n*= 496).

Parameter	NSP (*n* = 143)	ASP (*n* = 565)	Test value	*P*-value
Age	33.8 ± 4.7	35.4 ± 6.5	*U*= 35,624	0.0287
Semen volume (mL)	3.3 ± 1.4	3.2 ± 1.5	*U*= 38,758	0.4528
pH	7.3 ± 0.3	7.3 ± 0.3	*U*= 37,505	0.1608
Sperm concentration (10^6^ sperm/mL)	97.6 ± 61.4	59.3 ± 58.7	*U*= 22,743	<0.0001
Total sperm count (10^6^ sperm)	302.1 ± 231.7	181.0 ± 194.0	*U*= 22,068	<0.0001
Total motility (%)	57.4 ± 10.9	36.9 ± 16.2	*U*= 12,306	<0.0001
Vitality (%)	81.5 ± 7.7	69.5 ± 15.4	*U*= 19,714	<0.0001
Normal morphology (%)	5.3 ± 1.8	2.0 ± 1.5	*U*= 3962	<0.0001
Immature germ cells (10^6^ cells/mL)	1.00 ± 2.1	0.6 ± 1.4	*U*= 39,234	0.5709
Polymorphonuclear cells (10^6^ cells/mL)	0.1 ± 0.4	0.2 ± 1.2	*U*= 38,136	0.1388
Sperm DNA fragmentation (%)	8.3 ± 7.9	10.4 ± 10.3	*U*= 36,734	0.0857
Sperm chromatin decondensation (%)	5.5 ± 3.4	8.2 ± 6.2	*U*= 29,432	<0.0001
Absolute sORP (mV)	45.6 ± 27.7	49.3 ± 34.3	*U*= 38,328	0.3435
sORP Index (mV/10^6^ sperm/mL)	0.7 ± 0.7	2.8 ± 5.5	*U*= 24,330	<0.0001

Values are mean ± s.d.

ASP, abnormal sperm parameters; NSP, normal sperm parameters; *U*, Mann–Whitney test statistic value.

**Figure 1:**
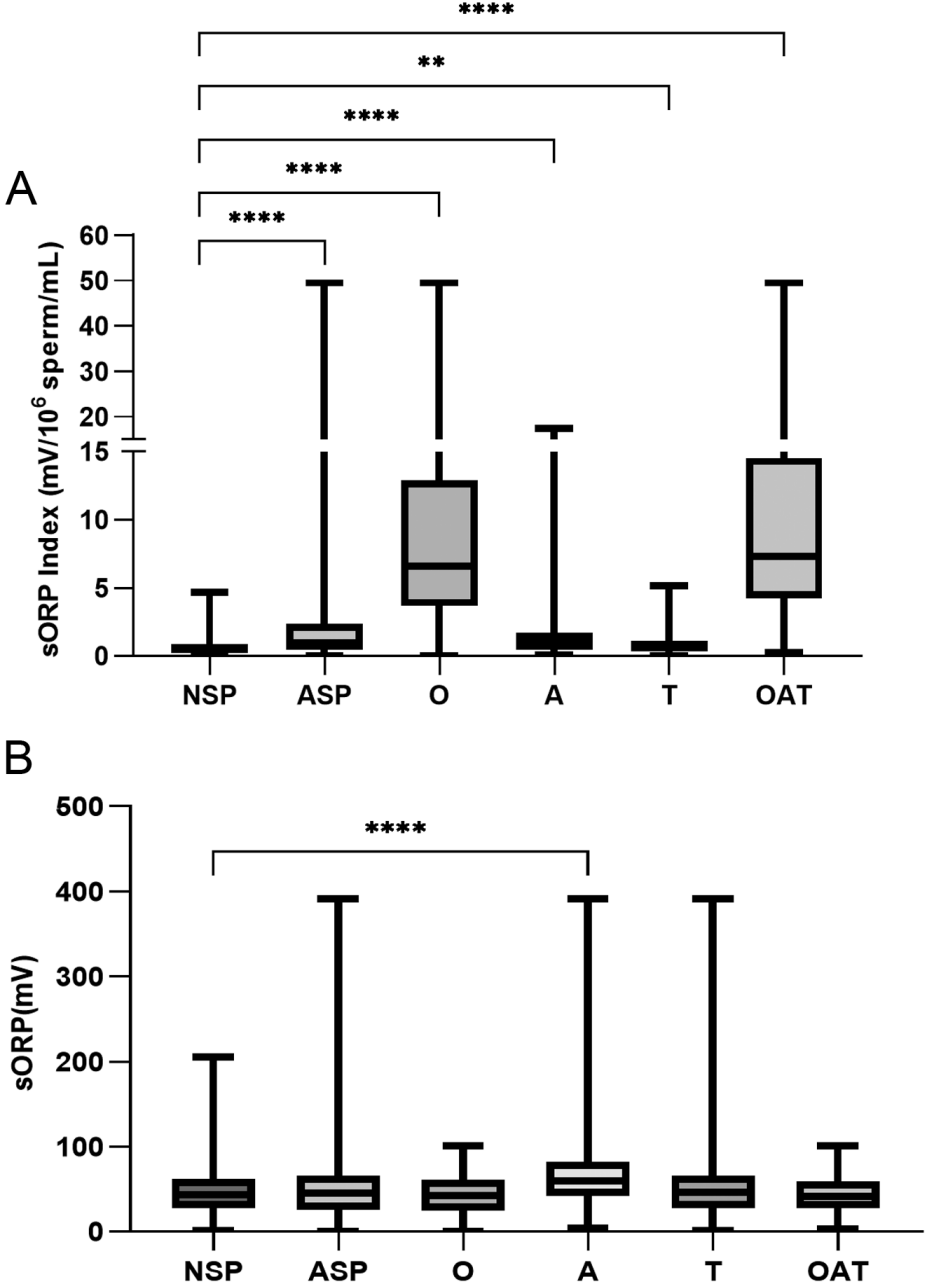
sORP index (mV/106 sperm/mL) and absolute sORP (mV) according to sperm parameters. Mann–Whitney *U* tests were performed between the NSP group and all the other groups. NSP, normal sperm parameters (*n* = 143); ASP, abnormal sperm parameters (*n* = 565); O, oligozoospermia (*n* = 115), ASP patients with sperm concentration <15 × 106 sperm/mL; A, asthenozoospermia (*n* = 320), ASP patients with sperm concentration ≥15 × 106 sperm/mL and motility <40%; T, teratozoospermia (*n* = 212), ASP patients with sperm concentration ≥15 × 106 sperm/mL, motility ≥40% but normal morphology <4%; OAT, oligoasthenoteratozoospermia (*n* = 78), ASP patients with sperm concentration <15 × 106 sperm/mL, motility <40% and normal morphology <4%. Box plot extremities represent minimum and maximum values.

ASP patients were grouped according to sperm parameters anomalies (oligozoospermia, asthenozoospermia and/or teratozoospermia). sORP index and absolute sORP were compared with NSP patients (Fig. 1). A significantly higher sORP index was found in oligozoospermic (*P*  < 0.0001), asthenozoospermic (*P*  < 0.0001), teratozoospermic (*P*  < 0.01) and oligoasthenoteratozoospermic (*P*  < 0.0001) patients. When sperm concentration was less than 15 × 10^6^sperm/mL (oligozoospermia and oligoasthenoteratozoospermia), sORP index was 13–15 times higher than that of the NSP group. In comparison, when motility was less than 40% (asthenozoospermia) and normal sperm morphology was inferior to 4%, sORP index was only 1.9 and 1.3 times, respectively, higher than that of the NSP patients (Fig. 1A). In comparison, sORP values were only significantly higher in asthenozoospermic patients (*P*  < 0.0001) (Fig. 1B). In addition, detailed descriptive data of sORP index and absolute sORP values for all patient groups showed the distribution of the data by percentile (Supplementary Tables 1 and 2, respectively).

Spearman correlations were performed to evaluate the associations between sORP index, absolute sORP and sperm parameters. As shown in Supplementary Table 3, sORP index strongly negatively correlated (*P*  < 0.0001) with sperm concentration (*r* = −0.7833), total sperm count (*r* = −0.7074), total motility (*r* = −0.3646), vitality (*r* = −0.2768) and morphology (*r* = −0.3141). sORP index also showed positive correlations with semen volume (*r* = 0.0928; *P*  < 0.05), sperm chromatin decondensation (*r* = 0.1639; *P*  < 0.0001) and polymorphonuclear leukocytes (*r* = 0.1028; *P*  < 0.01). There was, however, no correlation between sORP index and patients’ age, sperm viscosity and pH. It is interesting to note that sORP index weakly negatively correlated with sperm DNA fragmentation (*r* = −0.1221; *P*  < 0.05) and immature germ cells (*r*= −0.1109; *P*  < 0.01). As for absolute sORP, weak negative correlations were also found with normal morphology (*r*= −0.07939; *P*  > 0.0344) and immature germ cells (*r*= −0.09247, *P*  > 0.0138).

ROC curves were generated for the sperm parameters that best correlated with sORP index and absolute sORP (Supplementary Fig. 4). ROC analysis for sORP index (Supplementary Fig. 4A) showed high area under the curve (AUC) when evaluating sperm parameters normality (AUC = 0.6964, *P*  < 0.0001), sperm concentration (AUC = 0.9338, *P*  < 0.0001), total sperm count (AUC = 0.6568, *P*  < 0.0001), total motility (AUC = 0.6683, *P*  < 0.0001), total motile sperm count (AUC = 0.8659, *P*  < 0.0001), vitality (AUC = 0.7041, *P*  < 0.0001), morphology (AUC = 0.6658, *P*  < 0.0001) and SDI (AUC = 0.7188, *P*  < 0.01) (Supplementary Fig. 4). ROC analysis revealed that a sORP index cut-off of 0.79 could differentiate a NSP patient from an ASP patient with 57.7% sensitivity, 73.1% specificity, 69.9% PPV and 61.5% NPV (Supplementary Table 4). Detailed ROC curves analysis values are shown in Supplementary Table 4. ROC curves were also generated for absolute sORP values (Fig. 4B) and showed low AUC and non-significant data (data not shown).

To further study the distribution of sORP index in patients with sperm concentration abnormalities, ASP patients were subdivided into groups according to their concentration (Supplementary Fig. 5). Patients with less than 5 × 10^6^ sperm/mL showed significantly higher sORP index compared to NSP, reaching 15.8 mV/10^6^ sperm/mL. At this concentration, 100% of the sORP indexes (*n*  = 50) were higher than cut-offs found in this study (0.79 mV/10^6^ sperm/mL) and in the literature (1.34 mV/10^6^ sperm/mL), the lowest sORP index being 1.38. In all oligozoospermic patients (*n*  = 115), 97% of the sORP indexes were greater than 0.79, and 92% of them shown an index greater than 1.34 (data not shown). It is interesting to note that the higher the sperm concentration, the lower the sORP index. When sperm concentration was greater than 50 × 10^6^ sperm/mL, it was no longer possible to observe a significant difference with the NSP group.

**Figure 2:**
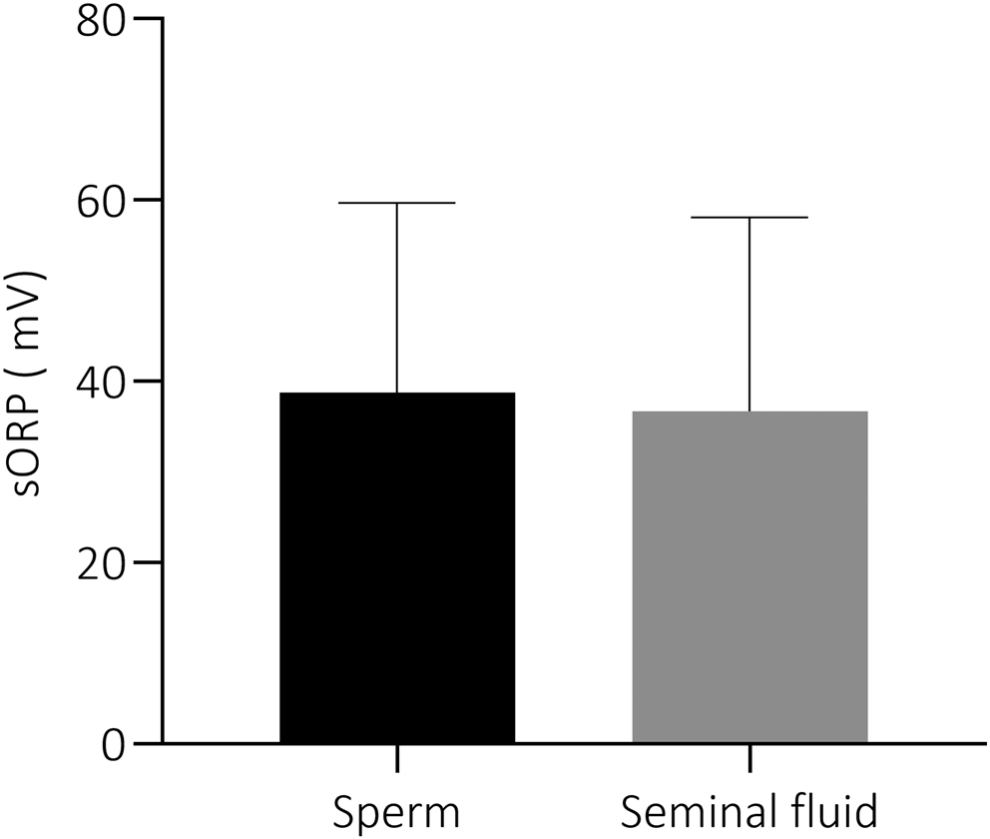
Differences of absolute sORP (mV) in sperm and seminal fluid alone. Sperm samples were centrifuged at high speed to obtain a sperm-free seminal fluid. sORP (mV) was measured in sperm and seminal fluid alone in each sperm sample. Mann–Whitney *U* test was performed between the two groups. *n*  = 57. Values are mean ± s.d.

In order to assess whether the correction method used to normalize the data did not induce a bias in the results obtained in this study, the contribution of spermatozoa in the establishment of the absolute sORP value by the MiOXSYS was investigated. Absolute sORP was measured in sperm and seminal fluid alone. No significant difference (*U*= 1524; *P* = 0.5694) was observed when spermatozoa were present in comparison to seminal fluid alone (Fig. 2). It is interesting to note that a strong decrease (more than 30%) was observed in 10 samples after sperm separation from the seminal fluid (difference of 30% to 96%). As shown in Supplementary Table 5, no difference was observed in age, sperm DNA integrity and sperm sORP between these 10 patients and the 47 others. However, sperm concentration was significantly higher (*U* = 101; *P = *0.0056) when the difference in sORP value after sperm separation was greater than or equal to 30% (Supplementary Table 5).

Since the presence of spermatozoa does not seem to affect the mV value given by the MiOXSYS, Spearman’s correlations were performed between absolute sORP and sperm parameters. Significant correlations were only found with the number of immature germ cells (*r*= −0.1449; *P*  < 0.001) (Table 2). No significant correlation was found with any of the other parameters. When investigating the association between sperm concentration and the other sperm parameters, significant correlations were found with total sperm count (*r*= 0.8743; *P*  < 0.0001), total motility (*r*= 0.4365; *P*  < 0.0001), vitality (*r*= 0.2848; *P*  < 0.0001), normal morphology (*r*= 0.3267; *P*  < 0.0001), number of polymorphonuclear leukocytes (*r*= 0.09251; *P*  < 0.05), sperm DNA fragmentation (*r*= 0.1379; *P*  < 0.001) and sperm chromatin decondensation (*r* = −0.2083; *P*  < 0.0001) (Table 2). Similar correlations were found when examining the association between total motility or normal morphology and the other semen parameters (Table 2). Not surprisingly, the Spearman’s *r* coefficients associated with sperm concentration were inversely similar to those observed for sORP index, in a sense that positive values are negative and vice versa. For example, the Spearman’s *r*coefficients of total motility, vitality, normal morphology or sperm chromatin decondensation index were −0.3646, −0.2768, −0.3141 or 0.1639 when looking at sORP index and 0.4365, 0.2848, 0.3267 or −0.1910 when looking at sperm concentration, respectively (Table 2).

**Table 2:** Spearman correlation coefficients of sORP index, sORP, sperm concentration, total motility and normal morphology with sperm parameters in all patients.

Parameter	sORP index	sORP	Sperm concentration	Total motility	Normal morphology
Total sperm count	−0.7074****	0.01546	0.8743****	0.4424****	0.3246****
Total motility	−0.3646****	−0.022	0.4365****	N/A	0.3846****
Vitality	−0.2768****	−0.06	0.2848****	0.5961****	0.2959****
Normal morphology	−0.3141****	−0.05025	0.3267****	0.3846****	N/A
Polymorphonuclear leukocytes	0.1028**	0.01698	−0.09251*	−0.1418***	−0.1288***
Immature germ cells	−0.1109**	−0.1449***	0.01597	−0.1167**	−0.02381
Sperm DNA fragmentation	−0.1221*	0.06949	0.1512***	−0.1061**	−0.044
Sperm chromatin decondensation	0.1639****	0.01021	−0.1910****	−0.3012****	−0.1831****
Absolute sORP	0.5086****	N/A	0.05476	−0.022	−0.05025
sORP index	N/A	0.5086****	−0.7833****	−0.3646****	−0.3141****

*P  < 0.05; **P  < 0.01; ***P  < 0.001; ****P  < 0.0001.

## Discussion

This study aimed to confirm the utility of the MiOXSYS in the context of male infertility investigation. MiOXSYS technology evaluates the oxido-reduction potential present in sperm samples and sORP absolute values represent a potential difference expressed in millivolts (mV) while the sORP index report this data on the sperm concentration (mV/10^6^ sperm/mL). Sperm samples from patients going to the Fertilys fertility center were analyzed and grouped according to the WHO 2010 sperm parameters reference values (World Health Organization 2010). Significant decreases (*P*  < 0.0001) in sperm concentration, total sperm count, motility, vitality and normal morphology were observed in ASP patients, when compared to NSP patients, as expected. Routinely analyzed in the Fertilys laboratory, DNA fragmentation and chromatin decondensation were evaluated in ASP and NSP patients. A significant difference was observed between the two groups, DNA integrity of ASP patients being more affected. sORP index was significantly higher (*P*  < 0.0001) in ASP patients, as previously described in recent literature (Agarwal & Wang 2017, Agarwal *et al.* 2017, 2018*a*, *b*, 2019, Ahmad *et al.* 2017, Arafa *et al.* 2018, Homa *et al.* 2019, Majzoub *et al.* 2020). Previous studies have shown significant correlations between sORP index and sperm parameters such as sperm concentration, total sperm count, total motility and normal morphology (Agarwal *et al.* 2017, Homa *et al.* 2019, Majzoub *et al.* 2020). Similar significant correlations between sORP index and sperm parameters were observed in this study. A significant positive correlation (*P*  < 0.01) was found between the polymorphonuclear leukocytes concentration and sORP index. This result was surprising considering that of Homa *et al.* who showed in 2019 that sORP index was not different between patients with elevated polymorphonuclear leukocytes and NSP patients (Homa *et al.* 2019). However, absolute sORP did not correlate with sperm parameters such as concentration or motility and only correlated with normal morphology and immature germ cells.

On the contrary, there was a significant negative correlation (*P*  < 0.01) between immature germ cells concentration and sORP index. This result would therefore mean that the fewer immature germ cells in the seminal plasma, the higher the sORP index. However, it is already known that immature germ cells are sources of oxidative stress (Ollero *et al.* 2001, Sabeti *et al.*2016, Barati *et al.* 2020). Sperm DNA fragmentation also negatively correlated with sORP index, which contradicts the established fact that oxidative stress can induce DNA damage (Wright *et al.* 2014, Jeng *et al.* 2015, William *et al.* 2015, Bisht *et al.*2017). This could be the result of a particular distribution of patients in our cohort regarding sperm DNA fragmentation, but it could also indicate that the MiOXSYS may not be suitable to associate sORP with DNA damage evaluated with the TUNEL method. Moreover, there was no correlation between absolute sORP values and DNA fragmentation or SDI. SDI ROC analysis has shown that a sORP index cut-off >1.54 mV/10^6^ sperm/mL indicates an NPV of 99.2% and a PPV of 5.5%, meaning that a sORP index below this cut-off will correctly identify 99.2% of patients with a normal SDI but will poorly identify the patients with an SDI >25%. These predictive values could be explained by the very low prevalence (2.3%) of SDI >25% in our population.

Over the years, different sORP thresholds have been proposed, with various sensitivity, specificity, NPV and PPV values (Agarwal *et al.* 2016, 2017, 2018*b*, Agarwal & Wang 2017, Arafa *et al.* 2018, Majzoub *et al.* 2020), the last proposed being >1.34 mV/10^6^ sperm/mL (Agarwal *et al.*2019). This cut-off can differentiate patients with NSP from patients with ASP with a sensitivity of 98.1%, a specificity of 40.6%, a PPV of 94.7% and an NPV of 66.6% (Agarwal *et al.* 2019). This means that this test will correctly identify not only 40.6% of the patients who do not have abnormal sORP but also 59.4% of patients having abnormal sORP when they do not. Moreover, in this study, we found a much lower threshold (>0.79 mV/10^6^ sperm/mL) and sensitivity, specificity, PPV and NPV values were quite different from the literature. For example, when comparing the values obtained by Agarwal *et al.* in 2019 with those found here, the established threshold was lower, the test was less sensitive but more specific and the PPV was similar but the NPV was reduced by half. The difference between the two thresholds can be explained partly by the sperm concentration in the ASP groups that was slightly different in the two studies. In Agarwal *et al.*, their average sperm concentration was 34.72 × 10^6^/mL, while in this present study, it was 59.3 × 10^6^/mL. So, for the same sORP value, dividing by a smaller concentration will result in a bigger sORP index. Moreover, in the context of male infertility investigation, the NPV and PPV values need to be higher since high PPV and NPV are needed to minimize false positives and negatives (Trevethan 2017).

In assisted reproductive treatments, spermatozoa need to be isolated from the seminal plasma in order to perform intra uterine insemination or *in vitro* fertilization, whether by a migration or filtration method or by the selection of mature sperm (Mehta & Sigman 2014). Clinicians need to know if there are high levels of oxidative stress in sperm, not necessarily in their environment. Ultimately, it is the sperm that will be used to fertilize the egg, *in vivo* or *in vitro*. However, the MiOXSYS does not seem to be able to give a measure of the oxidative stress in sperm but rather seems to measure the oxidation–reduction potential of the sperm environment, the seminal fluid. Furthermore, we observed a greater difference in absolute sORP after sperm separation in patients with higher sperm concentration. This finding suggests that the implication of sperm in the establishment of the absolute sORP value tends to be more likely seen at higher concentrations. At lower concentrations, the value given by the MiOXSYS would then represent the seminal fluid absolute sORP rather than the sperm absolute sORP. Knowing that seminal fluid has high or low oxido-reductive potential is interesting to understand in which environment the spermatozoa had developed, but more research needs to be done to associate the seminal fluid oxidative stress and the presence of oxidative stress directly in the spermatozoa.

In this study, we were able to demonstrate that the seminal fluid absolute sORP was not different from the one measured in the presence of spermatozoa, in agreement with the study published in 2016 by Agarwal *et al.* which demonstrated that the sORP index measured in seminal fluid was not different from the sORP index in sperm (Agarwal *et al.*2016). It is therefore possible to extrapolate that the absolute sORP was not different either. Establishing a ratio based on sperm concentration could then be questionable here. Furthermore, this would mean that the high sORP indexes found in oligozoospermic patients may not be the results of increased oxidative stress in their sperms although it has already been shown that oligozoospermic patients are subject to increased levels of oxidative stress (William *et al.* 2015) and reduced total antioxidant capacity (Colagar *et al.* 2013). When looking at the distribution of sORP indexes in ASP patients, we observed that when sperm concentration was less than 5 × 10^6^ sperm/mL, sORP indexes were the highest. However, when looking at sORP indexes of patients with higher sperm concentrations, we observed a drastic decrease in the average sORP index. Finally, when the concentration was greater than 50 × 10^6^ sperm/mL, we were no longer able to even observe a significant difference with the NSP group. In this case, it is possible that the ratio correction method ‘absolute sORP (mV)/sperm concentration (10^6^ sperm/mL)’ used here to normalize the data overestimate the sORP index in patients with low sperm concentration.

The ratio correction method is generally used in order to remove the effect of a confounding variable (in this case, sperm concentration) by normalizing the data using a ratio. However, in some cases, this method has the opposite effect, and the ratio can become inversely dependent on the confounding variable: when the confounding variable increases, the ratio then decreases (Karp *et al.* 2012). This phenomenon is observed in this study as well as in recent literature (Agarwal & Wang 2017, Agarwal *et al.* 2017, Arafa *et al.* 2018, Homa *et al.* 2019, Majzoub *et al.* 2020). This might then explain why the correlation coefficients of sORP index and sperm concentration with sperm parameters are inversely distributed. In 2017, Agarwal *et al.* already discussed about the importance to normalize sORP (mV) against sperm concentration. In doing so, sperm samples from men of various backgrounds (fertile, infertile, etiological conditions, geographical locations, etc.) could be compared (Agarwal *et al.* 2017). The authors also mentioned that sORP is not only dependent on the quantity but also on the quality of the spermatozoa. Since sperm is the principal source of ROS, the more poor-quality sperm, the higher the sORP. It actually makes sense to want to standardize absolute sORP on sperm concentration based on these arguments. However, in this case, the fact that the sperm concentration seems to be a confounding factor does not allow us to conclude that the sORP index is the correct ratio correction method for the study of sperm sORP in the context of male infertility.

The predictive values of ROC tests and the power of prediction of sORP index on sperm parameters normality remain debatable. In this study, we have shown that spermatozoa do not seem to have any impact on the establishment of the absolute sORP by the MiOXSYS. The promising results found in the literature would rather be linked to the denominator of the sORP index ratio, sperm concentration. However, it could be interesting to further investigate the comparisons between sperm absolute sORP and seminal fluid absolute sORP to determine if significant differences are still observed in some patients and to compare them with others already established methods measure the levels of ROS directly in spermatozoa such as the CellRox™ probes (Invitrogen) for example (Celeghini *et al.*2021).

## Supplementary Material

Supplementary Material

## Declaration of interest

Pierre Miron is the owner, CEO and president of Fertilys Inc. All the other authors have no conflict of interest to declare.

## Funding

This study did not receive any specific grant from any funding agency in the public, commercial, or not-for-profit sector.

## Authors contribution statement

F J participated in the study design, performed experiments, analysed data, and wrote the paper. C D performed experiments, analysed data and revised this manuscript. J L analysed data. M C B, C W X, S A, M B and P M participated in the study design.

## References

[bib1] Agarwal A Wang SM 2017 Clinical relevance of oxidation-reduction potential in the evaluation of male infertility. Urology 104 84–89. (10.1016/j.urology.2017.02.016) 28214572

[bib2] Agarwal A Sharma R Roychoudhury S Du Plessis S Sabanegh E 2016 MiOXSYS: a novel method of measuring oxidation reduction potential in semen and seminal plasma. Fertility and Sterility 106 566.e10–573.e10. (10.1016/j.fertnstert.2016.05.013) 27260688

[bib3] Agarwal A Roychoudhury S Sharma R Gupta S Majzoub A Sabanegh E 2017 Diagnostic application of oxidation–reduction potential assay for measurement of oxidative stress: clinical utility in male factor infertility. Reproductive Biomedicine Online 34 48–57. (10.1016/j.rbmo.2016.10.008) 27839743

[bib4] Agarwal A Sharma R Henkel R Roychoudhury S Sikka SC du Plessis S Sarda YB Speyer C Nouh M Douglas C 2018a Cumene hydroperoxide induced changes in oxidation reduction potential in fresh and frozen seminal ejaculates. Andrologia 50 e12796. (10.1111/and.12796) 28294377

[bib5] Agarwal A Henkel R Sharma R Tadros NN Sabanegh E 2018b Determination of seminal oxidation–reduction potential (ORP) as an easy and cost-effective clinical marker of male infertility. Andrologia 50 e12914. (10.1111/and.12914) 29057493

[bib6] Agarwal A Panner Selvam MK Arafa M Okada H Homa S Killeen A Balaban B Saleh R Armagan A Roychoudhury S 2019 Multi-center evaluation of oxidation-reduction potential by the MiOXSYS in males with abnormal semen. Asian Journal of Andrology 21 565–569. (10.4103/aja.aja_5_19) 31006711 PMC6859659

[bib7] Ahmad G Agarwal A Esteves SC Sharma R Almasry M Al-Gonaim A AlHayaza G Singh N Al Kattan L Sannaa WM 2017 Ascorbic acid reduces redox potential in human spermatozoa subjected to heat-induced oxidative stress. Andrologia 49 e12773. (10.1111/and.12773) 28251671

[bib8] Aitken RJ 2017 Reactive oxygen species as mediators of sperm capacitation and pathological damage. Molecular Reproduction and Development 84 1039–1052. (10.1002/mrd.22871) 28749007

[bib9] Aitken RJ Baker MA Sawyer D 2003 Oxidative stress in the male germ line and its role in the aetiology of male infertility and genetic disease. Reproductive Biomedicine Online 7 65–70. (10.1016/s1472-6483(1061730-0) 12930576

[bib10] Arafa M Agarwal A Al Said S Majzoub A Sharma R Bjugstad KB AlRumaihi K Elbardisi H 2018 Semen quality and infertility status can be identified through measures of oxidation–reduction potential. Andrologia 50 e12881. (10.1111/and.12881) 28771782

[bib11] Barati E Nikzad H Karimian M 2020 Oxidative stress and male infertility: current knowledge of pathophysiology and role of antioxidant therapy in disease management. Cellular and Molecular Life Sciences 77 93–113. (10.1007/s00018-019-03253-8) 31377843 PMC11105059

[bib12] Benchaib M Braun V Lornage J Hadj S Salle B Lejeune H Guérin JF 2003 Sperm DNA fragmentation decreases the pregnancy rate in an assisted reproductive technique. Human Reproduction 18 1023–1028. (10.1093/humrep/deg228) 12721180

[bib13] Bisht S Faiq M Tolahunase M Dada R 2017 Oxidative stress and male infertility. Nature Reviews: Urology 14 470–485. (10.1038/nrurol.2017.69) 28508879

[bib14] Bungum M Humaidan P Axmon A Spano M Bungum L Erenpreiss J Giwercman A 2007 Sperm DNA integrity assessment in prediction of assisted reproduction technology outcome. Human Reproduction 22 174–179. (10.1093/humrep/del326) 16921163

[bib15] Celeghini ECC Alves MBR de Arruda RP de Rezende GM Florez-Rodriguez SA de Sá Filho MF 2021 Efficiency of CellROX deep red® and CellROX orange® fluorescent probes in identifying reactive oxygen species in sperm samples from high and low fertility bulls. Animal Biotechnology 32 77–83. (10.1080/10495398.2019.1654485) 31424334

[bib16] Chao H Xiyue C Dejiang P Qihui L Yuanfeng Z Lixia L Anchun C Zhengli C 2018 Is male infertility associated with increased oxidative stress in seminal plasma? A-meta analysis. Oncotarget 9 24494–24513. (10.18632/oncotarget.25075) 29849956 PMC5966266

[bib17] Chen Q Zhao JY Xue X Zhu GX 2019 The association between sperm DNA fragmentation and reproductive outcomes following intrauterine insemination, a meta analysis. Reproductive Toxicology 86 50–55. (10.1016/j.reprotox.2019.03.004) 30905832

[bib18] Colagar AH Karimi F Jorsaraei SGA 2013 Correlation of sperm parameters with semen lipid peroxidation and total antioxidants levels in astheno- and oligoasheno-teratospermic men. Iranian Red Crescent Medical Journal 15 780–785. (10.5812/ircmj.6409) 24616785 PMC3929810

[bib19] Cooper TG Noonan E von Eckardstein S Auger J Baker HWG Behre HM Haugen TB Kruger T Wang C Mbizvo MT 2010 World Health Organization reference values for human semen characteristics. Human Reproduction Update 16 231–245. (10.1093/humupd/dmp048) 19934213

[bib20] Deng C Li T Xie Y Guo Y Yang Q Liang X Deng C-H Liu G-H 2019 Sperm DNA fragmentation index influences assisted reproductive technology outcome: a systematic review and meta-analysis combined with a retrospective cohort study. Andrologia 516 e13263. (10.1111/and.13263) 30838696

[bib21] Dutta S Majzoub A Agarwal A 2019 Oxidative stress and sperm function: a systematic review on evaluation and management. Arab Journal of Urology 17 87–97. (10.1080/2090598X.2019.1599624) 31285919 PMC6600059

[bib22] Griveau JF Le Lannou D 1997 Reactive oxygen species and human spermatozoa: physiology and pathology. International Journal of Andrology 20 61–69. (10.1046/j.1365-2605.1997.00044.x) 9292315

[bib23] Henkel R Hajimohammad M Stalf T Hoogendijk C Mehnert C Menkveld R Gips H Schill WB Kruger TF 2004 Influence of deoxyribonucleic acid damage on fertilization and pregnancy. Fertility and Sterility 81 965–972. (10.1016/j.fertnstert.2003.09.044) 15066449

[bib24] Homa ST Vassiliou AM Stone J Killeen AP Dawkins A Xie J Gould F Ramsay JWA 2019 A comparison between two assays for measuring seminal oxidative stress and their relationship with sperm DNA fragmentation and semen parameters. Genes 10 236. (10.3390/genes10030236) PMC6471935 30893955

[bib25] Jecht EW Poon CH 1975 Preparation of sperm-free seminal plasma from human semen. Fertility and Sterility 26 1–5. (10.1016/s0015-0282(1640868-x) 1109935

[bib26] Jeng HA Pan CH Chao MR Lin WY 2015 Sperm DNA oxidative damage and DNA adducts. Mutation Research: Genetic Toxicology and Environmental Mutagenesis 794 75–82. (10.1016/j.mrgentox.2015.09.002) 26653986 PMC4680842

[bib27] Jin SK Yang WX 2017 Factors and pathways involved in capacitation: how are they regulated? Oncotarget 8 3600–3627. (10.18632/oncotarget.12274) 27690295 PMC5356907

[bib28] Karp NA Segonds-Pichon A Gerdin AKB Ramírez-Solis R White JK 2012 The fallacy of ratio correction to address confounding factors. Laboratory Animals 46 245–252. (10.1258/la.2012.012003) 22829707 PMC4152922

[bib29] Lalkhen AG McCluskey A 2008 Clinical tests: sensitivity and specificity. Continuing Education in Anaesthesia Critical Care and Pain 8 221–223. (10.1093/bjaceaccp/mkn041)

[bib30] Li J Fine JP Safdar N 2007 Prevalence-dependent diagnostic accuracy measures. Statistics in Medicine 26 3258–3273. (10.1002/sim.2812) 17212380

[bib31] Majzoub A Arafa M Mahdi M Agarwal A Al Said S Al-Emadi I El Ansari W Alattar A Al Rumaihi K Elbardisi H 2018 Oxidation–reduction potential and sperm DNA fragmentation, and their associations with sperm morphological anomalies amongst fertile and infertile men. Arab Journal of Urology 16 87–95. (10.1016/j.aju.2017.11.014) 29713539 PMC5922185

[bib32] Majzoub A Arafa M El Ansari W Mahdi M Agarwal A Al-Said S Elbardisi H 2020 Correlation of oxidation reduction potential and total motile sperm count: its utility in the evaluation of male fertility potential. Asian Journal of Andrology 22 317–322. (10.4103/aja.aja_75_19) 31339113 PMC7275803

[bib33] Mehta A Sigman M 2014 Identification and preparation of sperm for art. Urologic Clinics of North America 41 169–180. (10.1016/j.ucl.2013.08.005) 24286775

[bib35] Ollero M Gil-Guzman E Lopez MC Sharma RK Agarwal A Larson K Evenson D Thomas AJ Alvarez JG 2001 Characterization of subsets of human spermatozoa at different stages of maturation: implications in the diagnosis and treatment of male infertility. Human Reproduction 16 1912–1921. (10.1093/humrep/16.9.1912) 11527898

[bib34] Pasqualotto Agarwal A 2000 Relationship between oxidative stress, semen characteristics, and clinical diagnosis in men undergoing infertility investigation. Fertility and Sterility 73 459–464. (10.1016/S0015-0282(9900567-1) 10688996

[bib36] Rael LT Bar-Or R Kelly MT Carrick MM Bar-Or D 2015 Assessment of oxidative stress in patients with an isolated traumatic brain injury using disposable electrochemical test strips. Electroanalysis 27 2567–2573. (10.1002/elan.201500178)

[bib37] Sabeti P Pourmasumi S Rahiminia T Akyash F Talebi AR 2016 Etiologies of sperm oxidative stress. International Journal of Reproductive Biomedicine 14 231–240. (10.29252/ijrm.14.4.231) 27351024 PMC4918773

[bib38] Steinberg DM Fine J Chappell R 2009 Sample size for positive and negative predictive value in diagnostic research using case-control designs. Biostatistics 10 94–105. (10.1093/biostatistics/kxn018) 18556677 PMC3668447

[bib39] Tomsu M Sharma V Miller D 2002 Embryo quality and IVF treatment outcomes may correlate with different sperm comet assay parameters. Human Reproduction 17 1856–1862. (10.1093/humrep/17.7.1856) 12093852

[bib40] Trevethan R 2017 Sensitivity, specificity, and predictive values: foundations, pliabilities, and pitfalls in research and practice. Frontiers in Public Health 5 307. (10.3389/fpubh.2017.00307) 29209603 PMC5701930

[bib41] Ullah Khan A Wilson T 1995 Reactive oxygen species as cellular messengers. Chemistry and Biology 2 437–445. (10.1016/1074-5521(9590259-7) 9383446

[bib42] William A Krishnan V Gafoor C A Apoorva M Banu C H Roy D D 2015 Evidence of increased oxidative stress and DNA damages in oligospermia. International Journal of Scientific and Engineering Research 6 747–751.

[bib43] World Health Organization 2010 Laboratory Manual for the Examination and Processing of Human Semen. WHO.

[bib44] Wright C Milne S Leeson H 2014 Sperm DNA damage caused by oxidative stress: modifiable clinical, lifestyle and nutritional factors in male infertility. Reproductive Biomedicine Online 28 684–703. (10.1016/j.rbmo.2014.02.004) 24745838

