# Supplementary material for: Reassessing the interpretation of oxidation–reduction potential in male infertility
Source: Reprod Fertil. 2022 Mar 18;3(2):67–76. doi: 10.1530/RAF-21-0005 (PMC9066566; doi:10.1530/RAF-21-0005)
Supplement: Supplementary Material [file supplementary_material.pdf]

SUPPLEMENTAL DATA

Supplemental table I. sORP index detailed descriptive statistics according to the patient anomaly groups. NSP: Normal Sperm Parameters (n=143); ASP: Abnormal Sperm Parameters (n=565); O: Oligozoospermia (n=115), ASP patients with sperm concentration < 15 x 106 sperm/mL; A: Asthenozoospermia (n=320), ASP patients with sperm concentration ≥ 15 x 106 sperm/mL and motility < 40%; T: Teratozoospermia (n=212), ASP patients with sperm concentration ≥ 15 x 106 sperm/mL, motility ≥ 40% but normal morphology < 4%; OAT: Oligoasthenoteratozoospermia (n=78), ASP patients with sperm concentration < 15 x 106 sperm/mL, motility < 40% and normal morphology < 4%.

	**Mean**	**Min-Max**	**2,5th**	**5th**	**95% CI 5th**	**10th**	**25th**	**50th Median**	**75th**	**99th**
**NSP (n=143)**	0.68	0.03-4.69	0.044	0.068	0.058 - 0.100	0.130	0.238	0.445	0.875	4.081
**ASP (n=565)**	2.81	0.01-49.42	0.084	0.140	0.116 - 0.147	0.220	0.458	1.000	2.373	28.530
**O (n=115)**	9.64	0.02-49.42	0.146	0.908	0.463 - 1.214	1.628	3.650	6.580	12.900	48.590
**A (n=320)**	1.27	0.04-17.32	0.090	0.130	0.106 - 0.146	0.217	0.438	0.900	1.698	6.161
**T (n=212)**	0.93	0.01-5.14	0.067	0.120	0.094 - 0.142	0.190	0.333	0.565	1.150	4.670
**OAT (78)**	10.88	0.25-49.42	1.020	1.695	0.992 - 2.153	2.338	4.235	7.295	14.540	49.420

Supplemental table II. Absolute sORP values detailed descriptive statistics according to the patient anomaly groups. NSP: Normal Sperm Parameters (n=143); ASP: Abnormal Sperm Parameters (n=565); O: Oligozoospermia (n=115), ASP patients with sperm concentration < 15 x 106 sperm/mL; A: Asthenozoospermia (n=320), ASP patients with sperm concentration ≥ 15 x 106 sperm/mL and motility < 40%; T: Teratozoospermia (n=212), ASP patients with sperm concentration ≥ 15 x 106 sperm/mL, motility ≥ 40% but normal morphology < 4%; OAT: Oligoasthenoteratozoospermia (n=78), ASP patients with sperm concentration < 15 x 106 sperm/mL, motility < 40% and normal morphology < 4%.

	**Mean**	**Min-Max**	**2,5th**	**5th**	**95% CI 5th**	**10th**	**25th**	**50th Median**	**75th**	**99th**
**NSP (n=143)**	45.56	0.90-204.90	3.40	7.34	5.13 - 10.43	14.60	27.20	42.80	61.90	160.70
**ASP (n=565)**	48.56	0.06-391.40	2.74	8.15	6.15 - 8.46	13.55	25.28	44.75	65.73	147.20
**O (n=115)**	44.57	0.20-100.40	1.56	6.26	3.62 - 9.98	12.92	24.10	41.90	61.20	100.20
**A (n=320)**	62.65	3.70-391.40	11.08	17.04	13.57 - 20.90	25.84	41.80	59.50	82.00	223.00
**T (n=212)**	49.66	0.90-391.40	7.00	10.70	9.01 - 11.18	15.09	27.55	46.25	66.00	144.40
**OAT (78)**	44.40	2.40-100.40	7.86	11.58	7.50 - 16.30	17.55	27.45	41.05	59.00	100.40

Supplemental table III. Spearman correlations of sORP index and absolute sORP with sperm parameters in all patients.

	sORP index		*Absolute sORP*	
Parameter	*Correlation coefficient*	*p-value*	*Correlation coefficient*	*p-value*
Age	0.04505	0.2312	0.03848	0.3065
pH	0.04598	0.2221	0.02269	0.5469
Viscosity	0.0006736	0.9857	-0.04173	0.2678
Semen volume	0.0928	**0.0135**	-0.07008	0.0624
Sperm concentration	-0.7833	**< 0.0001**	0.05940	0.1138
Total sperm count	-0.7074	**< 0.0001**	0.03117	0.4069
Total motility	-0.3646	**< 0.0001**	-0.03723	0.3286
Progressive motility	-0.8676	**0.0218**	-0.01483	0.6956
Total motile sperm count	-0.2852	**<0.0001**	-0.004382	0.9073
Vitality	-0.2768	**< 0.0001**	-0.06797	0.0703
Normal morphology	-0.3141	**< 0.0001**	-0.07939	**0.0344**
Polymorphonuclear leukocytes	0.1028	**0.0062**	-0.02894	0.4420
Immature germ cells	-0.1109	**0.0031**	-0.09247	**0.0138**
Sperm DNA fragmentation	-0.1221	**0.0027**	0.01325	0.7249
Sperm chromatin decondensation	0.1639	**< 0.0001**	0.05649	0.1327
Absolute sORP	0.5086	**< 0.0001**	-	-

Supplemental table IV. ROC analysis values of the sperm parameters that best correlate with sORP index. The Youden's index was used to identify the best sensitivity and specificity values. PPV and NPV were calculated according to the method from Steinberg *et al.* 2009 for each sperm parameter. SDI: Sperm Decondensation Index; NSP: Normal Sperm Parameters and ASP: Abnormal Sperm Parameters; PPV: Positive Predictive Value; NPV: Negative Predictive Value.

Parameter	*Area under curve*	*95% confidence interval*	*Cut-off*	*Sensitivity*	*Specificity*	*Prevalence*	*PPV*	*NPV*	*p-value*
Sperm parameters normality	0.6964	0.6519 to 0.7409	> 0.79	57.7%	73.1%	81%	90.1%	28.8%	< 0.0001
Concentration	0.9338	0.8999 to 0.9677	> 2.34	89.0%	91.2%	16.2%	66.2%	97.7%	< 0.0001
Total sperm count	0.6568	0.5990 to 0.7146	>1.28	57.4%	68.1%	16.4%	74.3%	11.0%	< 0.0001
Total motility	0.6683	0.6281 to 0.7084	> 0.81	66.9%	60.8%	45.2%	58.5%	69.0%	< 0.0001
Progressive motility	0.5383	0.4952 to 0.5814	>0.87	52.6%	54.1%	44.1%	47.5%	59.1%	0.0821
Total motile sperm count	0.8659	0.8290 to 0.9027	>1.89	73.1%	89.1%	23.6%	32.6%	8.5%	< 0.0001
Vitality	0.7041	0.6512 to 0.7569	> 0.81	79.7%	53.5%	16.0%	24.6%	93.2%	< 0.0001
Normal morphology	0.6658	0.6226 to 0.7090	< 45.25	99.1%	49.0%	71.8%	83.2%	95.7%	< 0.0001
SDI	0.7188	0.5833 to 0.8542	> 1.54	75.0%	70.4%	2.3%	5.5%	99.2%	0.0027

Supplemental table V. Comparisons of age, sperm DNA integrity and absolute sORP (mV) between patients having a strong decrease in absolute sORP and all of the other patients after sperm separation. Parameters were compared between the 10 patients having a difference of 30% and more in absolute sORP value after sperm separation and the 47 other patients. The minimum and maximum values are shown in brackets. *t*: Student *t* test statistic value. *U*: Mann-Whitney test statistic value. SDI: Sperm Decondensation Index.

Parameter	*All patients (n=57)*	*Difference of 30% and more (n=10)*	*Difference of less than 30% (n=47)*	*Test value*	*p-value*
Age	35.84 ± 6.39 (24-55)	35.6 ± 5.78 (27-44)	35.89 ± 6.58 (24-55)	*t*=0.1281	0.8985
Sperm concentration	92.42 ± 65.91 (6.5-316)	145.2 ± 70.6 (64.5-316)	80.7 ± 59.49 (6.5-263.5)	*U*=101	0.0056
Sperm DNA fragmentation	14 ± 10.21 (3-55)	14.5 ± 10.08 (3-32)	13.89 ± 10.35 (3-55)	*U*=217	0.8672
SDI	6.091 ± 4.29 (0-17)	6.6 ± 5.19 (1-16)	5.98 ± 4.12 (0-17)	*t*=0.4119	0.6820
Sperm sORP	38.75 ± 20.94 (0.3-99.4)	30.24 ± 10.87 (12.8-43.3)	40.56 ± 22.18 (0.3-99.4)	*t*=1.376	0.1743
Seminal plasma sORP	36.71 ± 21.37 (0.9-94.7)	15.55 ± 9.41 (0.9-28.6)	41.21 ± 20.5 (1.8-94.7)	*t*=3.782	0.0004

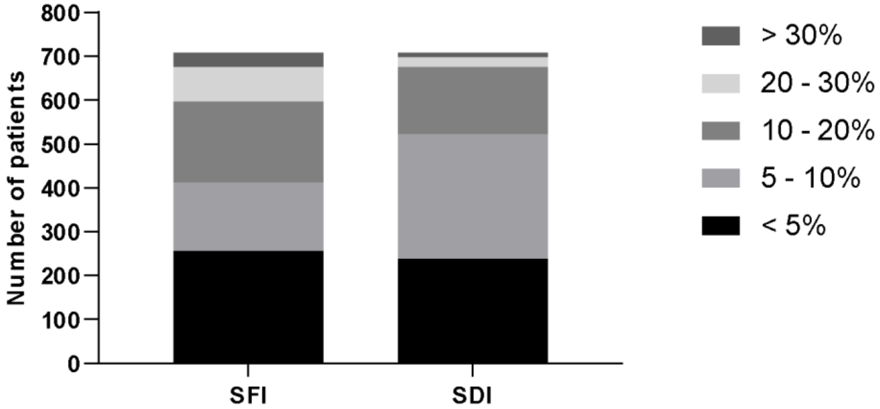

Supplemental figure 1. Distribution of patients according to their SDI or SFI. SDI: Sperm decondensation index; SFI: Sperm DNA fragmentation index.

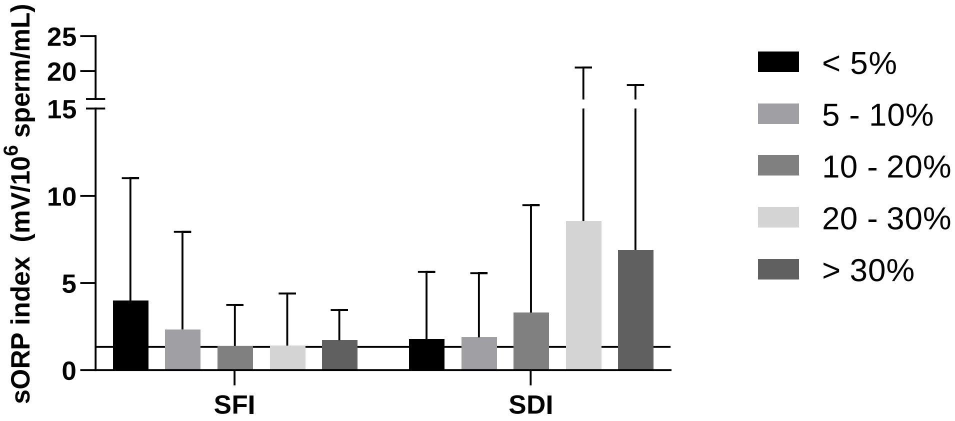

Supplemental figure 2. sORP indexes according to SDI or SFI. Data are mean ± standard deviation. SDI: Sperm decondensation index; SFI: Sperm DNA fragmentation index. The baseline represents the 1.34 sORP index cut-off value proposed by Agarwal *et al.* 2019. It is interesting to note that all sORP indexes were above this cut-off.

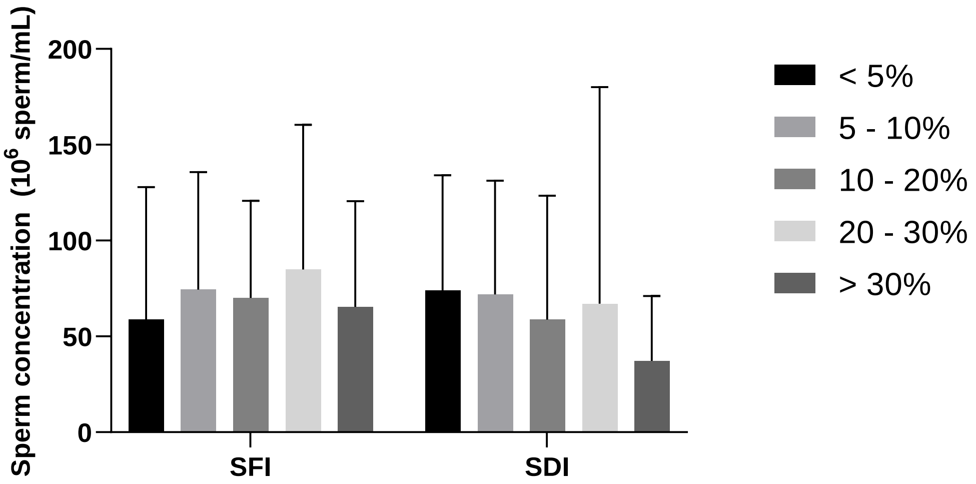

Supplemental figure 3. Sperm concentration according to SDI or SFI. Data are mean ± standard deviation. SDI: Sperm decondensation index; SFI: Sperm DNA fragmentation index.

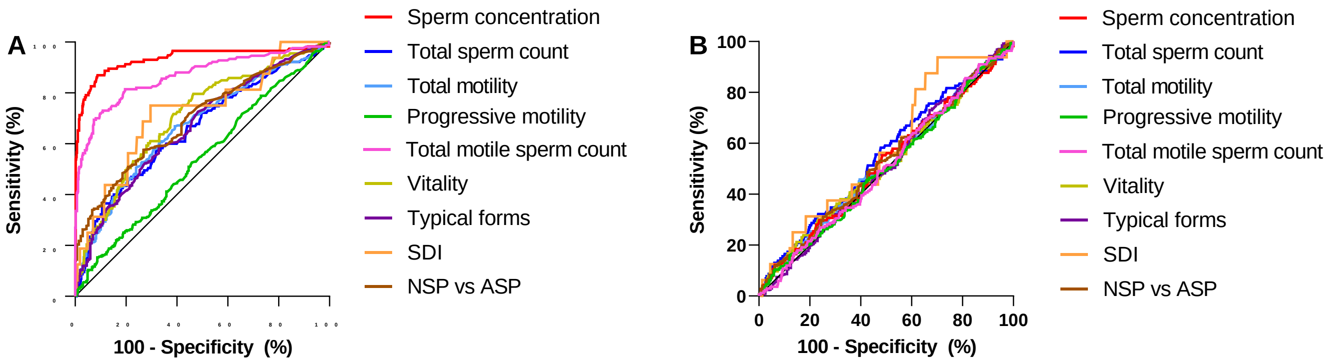

Supplemental figure 4. ROC curves showing sensitivity and specificity for sperm parameters that are best correlated with (A) sORP index and (B) absolute sORP. ROC curves were used to establish the best sORP index cut-off that would differentiate normal and abnormal sperm parameters. The Youden's index was used to identify the best sensitivity, specificity, positive predictive value and negative predictive value for each sperm parameter. The black line corresponds to the line of identity. SDI: Sperm Decondensation Index; NSP: Normal Sperm Parameters and ASP: Abnormal Sperm Parameters.

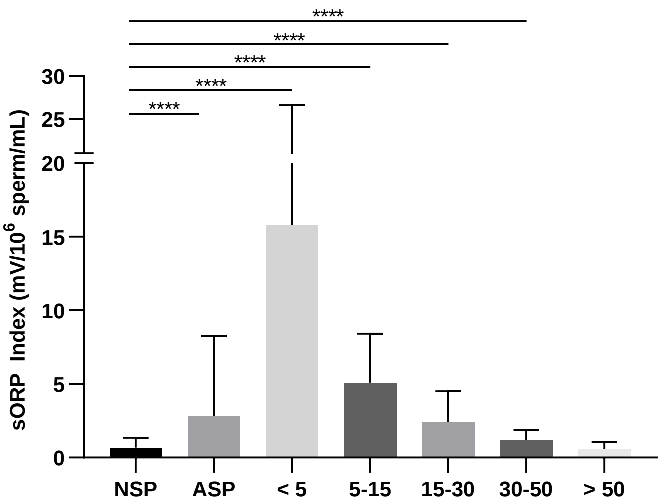

Supplemental figure 5. sORP index in ASP patients according to sperm concentration. Mann-Whitney U tests were performed between the NSP group and all the other groups. NSP: Normal Sperm Parameters (n=134); ASP: Abnormal Sperm Parameters (n=574); < 5: concentration < 5.10^6 sperm/mL (n=49); 5-15 : 5.10^6 sperm/mL < concentration < 15.10^6 sperm/mL (n=66); 15-30 : 15.10^6 sperm/mL < concentration < 30.10^6 sperm/mL (n=93); 30-50 : 30.10^6 sperm/mL < concentration < 50.10^6 sperm/mL (n=114); > 50 : concentration > 50.10^6 sperm/mL (n=252). Values are mean ± standard deviation. **** *p*<0.0001.
